# Molecular epidemiological tracing of a cattle rabies outbreak lasting less than a month in Rio Grande do Sul in southern Brazil

**DOI:** 10.1186/s13104-016-1898-5

**Published:** 2016-02-12

**Authors:** Takuya Itou, Toshiharu Fukayama, Nobuyuki Mochizuki, Yuki Kobayashi, Eduardo R. Deberaldini, Adolorata A. B. Carvalho, Fumio H. Ito, Takeo Sakai

**Affiliations:** Nihon University Veterinary Research Center, Nihon University, 1866 Kameino, Fujisawa, Kanagawa 252-0880 Japan; Department of Preventive Veterinary Medicine, Faculty of Agriculture and Veterinary Science, UNESP, Via de Acesso Prof. Paulo Donato Castellane, Jaboticabal, Sao Paulo, 14884-900 Brazil; Department of Preventive Veterinary Medicine and Animal Health, Faculty of Veterinary Medicine and Zootechny, University of São Paulo, Av. Prof. Dr. Orlando Marques de paiva, 87, Cidade Universtiátria, Sao Paulo, São Paulo 05508-000 Brazil

**Keywords:** Brazil, Cattle rabies, Molecular epidemiology, Vampire bat

## Abstract

**Background:**

Vampire bat-transmitted cattle rabies cases are typically encountered in areas where the disease is endemic. However, over the period of a month in 2009, an outbreak of cattle rabies occurred and then ended spontaneously in a small area of the Rio Grande do Sul State in southern Brazil. To investigate the epidemiological characteristics of this rabies outbreak in Rio Grande do Sul, 26 nucleotide sequences of rabies virus (RABV) genomes that were collected in this area were analyzed phylogenetically.

**Results:**

Nucleotide sequence identities of the nucleoprotein gene and G–L intergenic region of the 26 RABVs were greater than 99.6 %. Phylogenetic analysis showed that all RABVs clustered with the vampire bat-related cattle RABV strains and that the RABVs were mainly distributed in southern Brazil.

**Conclusions:**

The findings of the present study suggested that a small population of rabid vampire bats carrying a single RABV strain produced a spatiotemporally restricted outbreak of cattle rabies in southern Brazil.

**Electronic supplementary material:**

The online version of this article (doi:10.1186/s13104-016-1898-5) contains supplementary material, which is available to authorized users.

## Background

Rabies is a fatal infectious disease that causes encephalomyelitis. Genetic diversity among rabies viruses (RABVs) depends on the host species and its geographic distribution. Rabies is endemic in Brazil, and RABVs have been isolated from a variety of mammalian hosts, including dogs, foxes, cats, cattle, and hematophagous, insectivorous and frugivorous bats [1–3]. Vampire bats, particularly *Desmodus rotundus*, are important vectors of rabies in Latin America. Rabies transmission from vampire bats to humans occurs frequently, primarily in the Amazonian regions of Brazil and Peru, and cattle rabies transmitted by vampire bats is also common in Brazil [4]. Vampire bat-related rabies is thus a serious problem from both animal and public health standpoints.

Molecular and geographic epidemiological analyses of livestock rabies in northeastern, central and southeastern Brazil revealed that RABVs isolated from livestock were closely related to the virus strains found in vampire bat populations, and that the RABVs in these areas were stable within a spatiotemporal context [5]. However, relatively little information currently exists on the molecular epidemiology of RABVs isolated from livestock in southern Brazil.

The incidence of vampire bat-transmitted cattle rabies cases is typically restricted to areas where the disease is endemic [5–7]. However, between February and March 2009, an outbreak of cattle rabies occurred and ended spontaneously in a small region of Rio Grande do Sul State (RS) in southern Brazil. To investigate the epidemiological characteristics of this rabies outbreak in RS, the RABVs obtained in this area were subjected to phylogenetic analysis.

## Methods

**Fig. 1:**
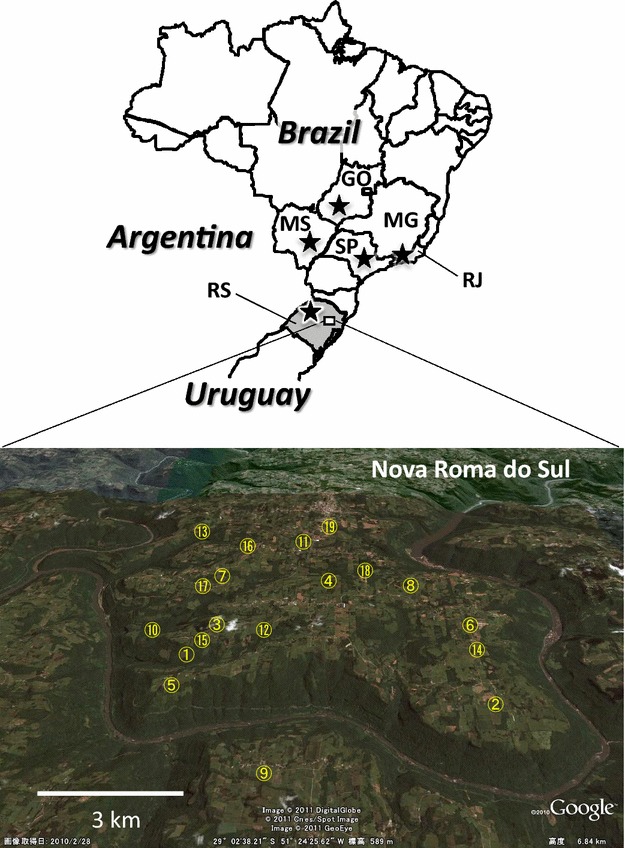
Location of farms infected with cattle rabies in Nova Roma do Sul, the state of Rio Grande do Sul (RS). *Numbers* on the map correspond with the site numbers given in Table 1. *Star-shaped* symbols indicate the states, where RABVs belonging to C-19 lineage were distributed [5]. Abbreviation states in Brazil are as follows: *GO* Goiás; *MG* Minas Gerais; *MS* Mato Grosso do Sul; *RJ* Rio de Janeiro; *SP* São Paulo

An outbreak of cattle rabies occurred in a hilly area (<5 km radius) in the city of Nova Roma do Sul in Rio Grande do Sul State, southern Brazil (Fig. 1). This outbreak occurred on a total of 19 cattle farms between February and March 2009, and ended spontaneously without any mitigation measures being implemented. Outbreaks of cattle rabies in this region had not been observed for at least 3 years before or after this outbreak. Brain specimens from these livestock were diagnosed as RABV-positive by an immunofluorescent antibody test and viral RNA was extracted from the brains of livestock using the QIAamp Viral RNA Mini Kit (Qiagen, Hilden, Germany). Nucleoprotein (N) gene sequences from the Brazilian RABV samples were amplified by RT-PCR and determined using applied biosystems 3130 genetic analyzer (ABI) using primers described previously [8, 9]. Information of primers used for RT-PCR and sequencing are shown in Additional file 1: Table S1. Multiple alignment for the datasets of N and G–L genes were made by MAFFT program [10]. Neighbor-joining tree of N gene with 1000 bootstrap replicates was constructed with the p-distance using MEGA ver 6.10 [11]. The nucleotide sequences retrieved from GenBank to construct the phylogenetic tree are listed in Additional file 2: Table S2.

The geographic origins of the RABV sequences obtained from the Brazilian livestock were plotted at the municipal level in the federal states in which infection was observed using MapInfo Professional GIS software (ver. 8.0, MapInfo Japan K.K., Tokyo, Japan) and Google Earth (ver.6.0, Google, Mountain View, CA, USA). Brazilian maps were obtained from Brasil em Relevo—Embrapa Monitoramento por Satélite [12].

## Results

A total of 26 RNA samples were extracted from brains of rabid cattle from the 19 farms surveyed in this study (Fig. 1; Table 1). The PCR amplified sequences of the 1424-nt N**-**mRNA region and 578-nt G–L region were determined for all 26 RABV samples [GenBank: AB685222–AB685247 and LC057591–LC057616] (Table 1). The results from N gene sequences showed that, with the exception of BRbv1289, the nucleotide and amino acid sequence identities of the RS RABVs were 100 %. Between BRbv1285, which was selected among the 25 identical RABV RNAs as the representative sequence, and BRbv1289, nucleotide and amino acid sequence identities were more than 99.9 and 100 %, respectively. To validate the genomic homogeneity among the RABV samples, the variable G–L intergenic 578-bp region (position: 4929–5514 of PV) was additionally analyzed. The nucleotide sequence identity was more than 99.6 % among all 26 RABVs, showing the quite high homogeneity of epidemic RABVs in this outbreak.

**Table 1:** Rabies virus samples obtained from the Rio Grande do Sul

Sample name	Date of sampling	Site of occurrence^b^	GenBank accession #	
			N gene	G–L intergenic
BRbv1285	28/02/2009	1	AB685222	LC057591
BRbv1286	08/03/2009	2	AB685223	LC057592
BRbv1287	18/03/2009	3	AB685224	LC057593
BRbv1288	28/02 2009	4	AB685225	LC057594
BRbv1289	28/02/2009	5	AB685226	LC057595
BRbv1290	28/02/2009	6	AB685227	LC057596
BRbv1291	23/02/2009	1	AB685228	LC057597
BRbv1292	11/03/2009	7	AB685229	LC057598
BRbv1293	13/03/2009	8	AB685230	LC057599
BRbv1294	06/03/2009	6	AB685231	LC057600
BRbv1297	08/03/2009	9	AB685232	LC057601
BRbv1298	09/03/2009	10	AB685233	LC057602
BRbv1299	12/03/2009	1	AB685234	LC057603
BRbv1301	18/02/2009	11	AB685235	LC057604
BRbv1302	13/03/2009	12	AB685236	LC057605
BRbv1303	18/03/2009	3	AB685237	LC057606
BRbv1304	18/03/2009	13	AB685238	LC057607
BRbv1305	23/02/2009	2	AB685239	LC057608
BRbv1306	–^a^	14	AB685240	LC057609
BRbv1307	12/03/2009	15	AB685241	LC057610
BRbv1308	27/02/2009	16	AB685242	LC057611
BRbv1309	02/03/2009	17	AB685243	LC057612
BRbv1310	23/02/2009	18	AB685244	LC057613
BRbv1311	25/02/2009	19	AB685245	LC057614
BRbv1312	28/02/2009	5	AB685246	LC057615
BRbv1313	04/03/2009	9	AB685247	LC057616

^a^No information

^b^Numbers correspond to those in Fig. 1

**Fig. 2:**
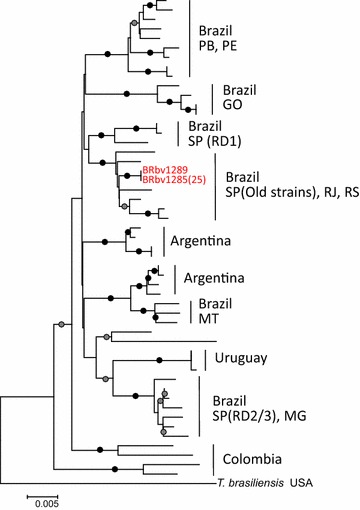
Phylogenetic tree based on a partial N gene sequence (corresponding to 203–1420 nts of PV strain) of RABV samples from southern Brazil and surrounding countries. *Black* and *gray* circles indicate the interior branches with bootstrap values >90 and 90–70 %, respectively. RABV samples in RS were indicated by red text. The *numbers in* *parenthesis* show the number of other strains with 100 % identical sequence to the indicated strain. Abbreviation states in Brazil are as follows: *GO* Goiás; *MG* Minas Gerais; *MT* Mato Grosso; *PB* Paraiba; *PE* Pernambico; *RJ* Rio de Janeiro; *SP* São Paulo. RABV sequence from *Tadarida brasiliensis* was used as outgroup

Phylogenetic analysis based on a partial N sequence (position: 203–1420 of PV) revealed that 26 RABV samples formed a monophyletic cluster in vampire bat-related RABV lineage (Fig. 2). Comparison with RABVs obtained from neighboring countries, indicated that these 26 RABVs formed a sub-cluster with RABVs collected in the states of Sao Paulo (SP) and Rio de Janeiro (RJ) in Brazil. In addition to this sub-cluster, there are two another sub-clusters consisted of RABV samples collected in the state of SP and neighboring areas. These lineages were named the lineages of RD1, RD2/3, and Old strains by Carnieli et al. [13]. RS strains belonged to the lineage of Old strains and shared a common ancestor with RD1 lineage. RABVs in RD1 and RD2/3 lineages were distributed in mountain range located in the boundary of the states of SP, Minas Gerais, and RJ, while Old strains were isolated from towns in the interior of the state of SP [13].

Using a 203-bp segment (position: 109–311 of PV) of the N gene, Kobayashi et al. reported the existence of at least 24 RABV genetic variants among the vampire bat-transmitted rabies cases in cattle in Brazil [5]. Using the same 203-bp segment, our analysis revealed that the 26 RS RABVs of the present study correlate with the C-19 lineage described by Kobayashi et al. [5] (Additional file 3: Figure S1). Samples belonging to the C-19 lineage were distributed in the states of RS, Goiás, Mato Grosso do Sul, SP and RJ, which are the southeastern regions of Brazil [5].

## Discussion

Nucleoprotein gene and G–L intergenic segments from the 26 RABV samples collected in early spring of 2009 from separate farms in a restricted region of Nova Roma do Sul, RS, were sequenced and subjected to phylogenetic analysis. The genetic analysis revealed that the nucleotide and amino acid sequences were almost identical among the 26 RABVs and shared a common ancestor, suggesting the RABV strain was transmitted from a small population of rabid vampire bats infected with RABV, which may be derived from a single introduction, to a cattle population grazing in Nova Roma do Sul.

Generally, vampire bat-transmitted RABVs were restricted to specific geographic areas because of the vampire bat ecology. The phylogenetic analysis described here revealed that all of the livestock RABVs in RS were derived from vampire bat rabies. Although we were unable to investigate the prevalence of rabid vampire bats in this area, the findings of the present study revealed that the 26 RABVs correspond with the “Old strains” lineage reported by Carnieli Jr et al. and C-19 lineage identified by Kobayashi et al. [5, 13]. The distribution of RABV lineages in Brazil is known to be delimited by geomorphological boundaries, such as mountain ranges and rivers [14]. The reason why the C-19 lineage consists of RABVs from Brazil and none from Argentina may be thus because a very large river, the Uruguay River, separates the Brazil and Argentina. However, the C-19 lineage is distributed over larger areas. Since the “Old strains” lineage, which was made up basically of the isolates collected before 1998, has been endemic in the state of SP before epizootic of RD2/RD3 were observed [13], suggesting that RABVs in Old strains lineages may have gradually spread in southern Brazil, including the state of RS.

The typical distribution range of *D. rotundas* activity around a roost is about 10 km^2^ [15]; the range of this outbreak was approximately 80 km^2^. In extensive random surveys conducted in Trinidad and Brazil, the prevalence of the virus among vampire bats in the field was 0.46–0.75 % (see Baer) [16]. Further, no rabies outbreaks have been reported in the past 3 years in this region, and the outbreak ended without any specific prevention measures being implemented for cattle rabies. Although the non-migratory, vampire bats have distribution ranges of between 10 and 20 km^2^ [17], it has been suggested that the vampire bats may need to fly over longer distances to feed and that they share roosts with other bat species; indeed, this may explain how the RABV could spread over a short period of time [18]. However, the cattle rabies cases described in the present study ended suddenly over the period of a month. There are several possible reasons why the activities of the vampire bats may have been limited and why this outbreak of cattle rabies ended suddenly. The affected area was geographically isolated by a river valley that may have limited the feeding activities and distribution of the bats. It is also possible that the size of the vampire bat population in the region may have been too small to act as reservoir for RABV.

Taken together, it is considered that this outbreak was caused by small population of vampire bats that were vectors of the RABV lineage that is distributed mainly in southern Brazil. It is considered that the relatively restricted range of bats contributed to the sudden end of the outbreak before it could spread to other regions.

## Conclusions

Segments of N gene and G–L intergenic region sequences from 26 RABV samples that were collected in early spring from separate farms in small region of Nova Roma do Sul, Rio Grande do Sul, in 2009, were subjected to phylogenetic analysis. The N gene and G–L region sequence analysis revealed that the 26 RABVs had almost identical nucleotide and amino acid sequences. The outbreak was likely caused by a small population of vampire bats that were infected with a single strain of the RABV lineage found mainly in southern Brazil that invaded the area of the study site.

## Additional files

10.1186/s13104-016-1898-5 Primers used for RT-PCR and sequencing.
10.1186/s13104-016-1898-5 Nucleotide sequences RABV N gene retrieved from GenBank.
10.1186/s13104-016-1898-5 Phylogenetic tree based on nucleoprotein gene 203 nts (corresponding 107–311 nts of PV strain) using Brazilian RABV isolates. The 24 lineages classified by Kobayashi et al. [5] and RS RABV samples in this study were compared. All RS samples belonged to C-19 lineage maintained in Southwestern Brazil. The symbols for each lineage correspond to the results by Kobayashi et al. [5].

## Abbreviation

RABV: rabies virus
