# Supplementary material for: Molecular epidemiological tracing of a cattle rabies outbreak lasting less than a month in Rio Grande do Sul in southern Brazil
Source: BMC Res Notes. 2016 Feb 12;9:87. doi: 10.1186/s13104-016-1898-5 (PMC4751707; doi:10.1186/s13104-016-1898-5)
Supplement: Supplementary file 3 — 10.1186/s13104-016-1898-5 Phylogenetic tree based on nucleoprotein gene 203 nts (corresponding 107–311 nts of PV strain) using Brazilian RABV isolates. The 24 lineages classified by Kobayashi et al. [5] and RS RABV samples in this study were compared. All RS samples belonged to C-19 lineage maintained in Southwestern Brazil. The symbols for each lineage correspond to the results by Kobayashi et al. [5]. [file 13104_2016_1898_MOESM3_ESM.pptx]

## Slide 1
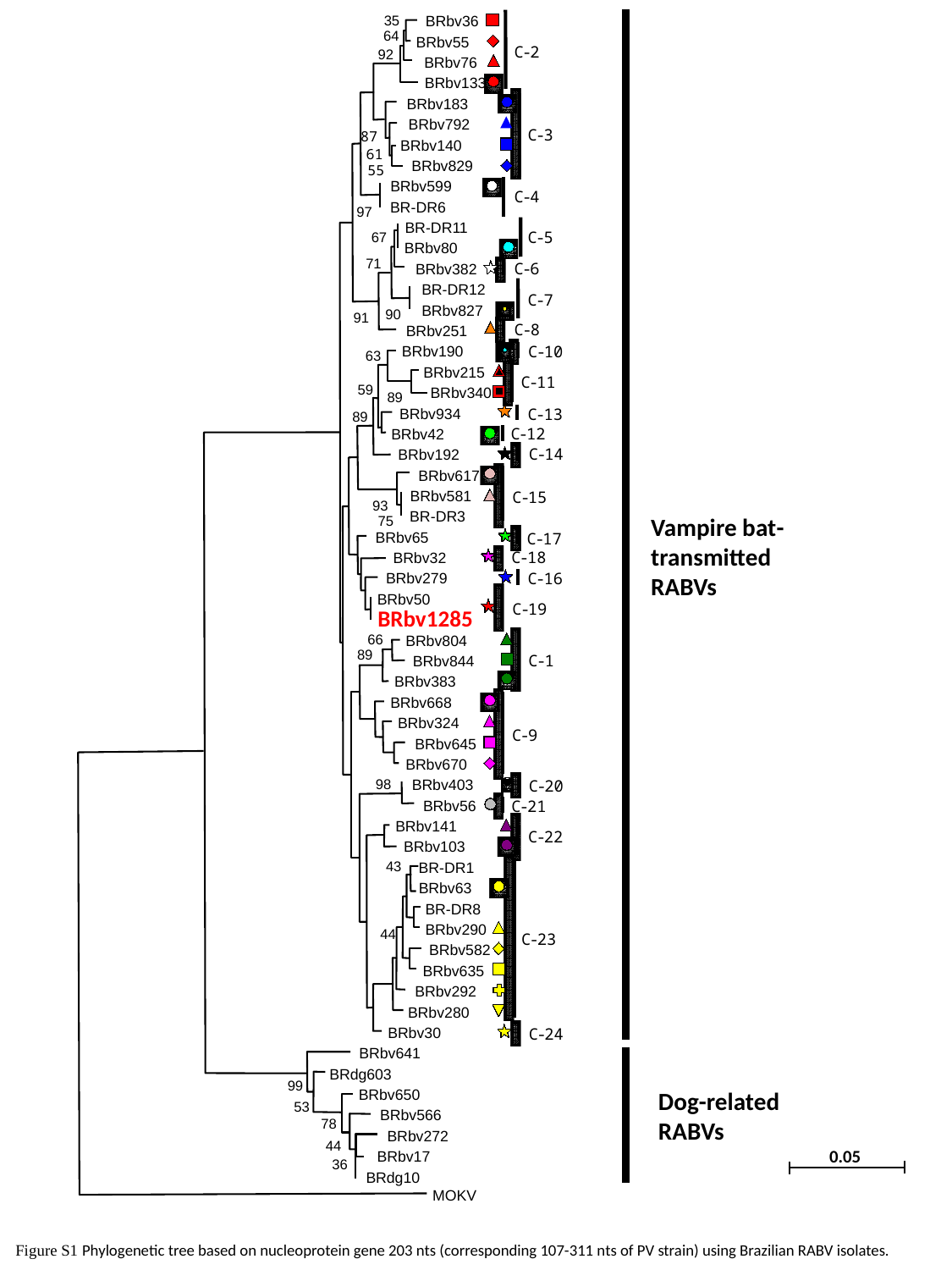

35
BRbv36
64
BRbv55
92
BRbv76
BRbv133
BRbv183
BRbv792
BRbv140
BRbv829
BRbv599
BR-DR6
97
BR-DR11
67
BRbv80
BRbv382
BR-DR12
BRbv827
90
BRbv251
BRbv190
BRbv215
BRbv340
BRbv934
BRbv42
BRbv192
BRbv617
BRbv581
93
BR-DR3
75
BRbv65
BRbv32
BRbv279
BRbv50
BRbv1285
BRbv804
BRbv844
BRbv383
BRbv668
BRbv324
BRbv645
BRbv670
98
BRbv403
BRbv56
BRbv141
BRbv103
BR-DR1
BRbv63
BR-DR8
BRbv290
BRbv582
BRbv635
BRbv292
BRbv280
BRbv30
BRbv641
BRdg603
99
BRbv650
53
BRbv566
78
BRbv272
44
BRbv17
36
BRdg10
MOKV
C
-
2
C
-
3
87
61
55
C
-
4
C
-
5
71
C
-
6
C
-
7
91
C
-
8
C
-
10
63
C
-
11
59
89
C
-
13
89
C
-
12
C
-
14
C
-
15
Vampire bat-transmitted RABVs
C
-
17
C
-
18
C
-
16
C
-
19
66
89
C
-
1
C
-
9
C
-
20
C
-
21
C
-
22
43
44
C
-
23
C
-
24
Dog-related RABVs
0.05
Figure S1 Phylogenetic tree based on nucleoprotein gene 203 nts (corresponding 107-311 nts of PV strain) using Brazilian RABV isolates.
